# Sodium glucose cotransporter 2 inhibitors: mechanisms of action in heart failure

**DOI:** 10.1007/s10741-020-10041-1

**Published:** 2020-11-04

**Authors:** Mieczysław Dutka, Rafał Bobiński, Izabela Ulman-Włodarz, Maciej Hajduga, Jan Bujok, Celina Pająk, Michał Ćwiertnia

**Affiliations:** 1 Faculty of Health Sciences, Department of Biochemistry and Molecular Biology, University of Bielsko-Biała, Willowa St. 2, 43-309 Bielsko-Biała, Poland; 2 Faculty of Health Sciences, Department of Emergency Medicine, University of Bielsko-Biała, Willowa St. 2, 43-309 Bielsko-Biała, Poland

**Keywords:** Heart failure, Cardiovascular diseases, Diabetes, Sodium-glucose cotransporter 2 inhibitors

## Abstract

Diabetes is a key independent risk factor in the development of heart failure (HF) and a strong, adverse prognostic factor in HF patients. HF remains the primary cause of hospitalisation for diabetics and, as previous studies have shown, when HF occurs in these patients, intensive glycaemic control does not directly improve the prognosis. Recent clinical studies assessing a new class of antidiabetic drugs, sodium-glucose cotransporter 2 inhibitors (SGLT2is) showed some unexpected beneficial results. Patients treated with SGLT2is had a significant decrease in both cardiovascular (CV) and all-cause mortality and less hospitalisations due to HF compared to those given a placebo. These significant clinical benefits occurred quickly after the drugs were administered and were not solely due to improved glycaemic control. These groundbreaking clinical trials’ results have already changed clinical practice in the management of patients with diabetes at high CV risk. These trials have triggered numerous experimental studies aimed at explaining the mechanisms of action of this unique group of drugs. This article presents the current state of knowledge about the mechanisms of action of SGLT2is developed for the treatment of diabetes and which, thanks to their cardioprotective effects, may, in the future, become a treatment for patients with HF.

## Introduction

A new group of antidiabetic drugs, sodium-glucose cotransporter 2 inhibitors (SGLT2is), has recently been shown to significantly improve the prognosis in high-risk cardiovascular (CV) patients with type 2 diabetes. The CV outcomes of SGLT2is (empagliflozin, canagliflozin and dapagliflozin) have been evaluated in, among others, three randomised clinical trials: Empagliflozin Cardiovascular Outcome Event Trial in Type 2 Diabetes Mellitus Patients (EMPA-REG OUTCOME), Canagliflozin Cardiovascular Assessment Study (CANVAS) and Dapagliflizin Effect on Cardiovascular Events Thrombosis in Myocardial Infarction 58 (DECLARE—TIMI 58) [1–10]. The first and thus groundbreaking EMPA-REG OUTCOME study showed that the use of empagliflozin in patients with type 2 diabetes and a high CV risk reduced CV mortality by 38%, without significantly reducing the number of non-fatal myocardial infarctions and strokes [11, 12]. The use of empagliflozin in this group of patients also resulted in a 35% reduction in hospitalisation due to heart failure (HF) and a 32% reduction in all-cause mortality [11, 12]. Such significant, beneficial effects of the antidiabetic drug, empagliflozin, cannot be fully explained by its antidiabetic, metabolic effect. The unique mechanism of action of this drug, whereby it inhibits SGLT2, also results in certain haemodynamic effects, which are beneficial from the point of view of CV diseases. It is these haemodynamic effects, such as lowering blood pressure or reducing extracellular fluid volume, that may be responsible, to a large degree, for improving the prognosis of high-risk CV patients treated with SGLT2is [1]. However, even if these beneficial haemodynamic effects of SGLT2is are taken into account, it does not fully explain the beneficial CV effects of using these drugs. It is also known that SGLT2 is not present in the heart of humans or other mammals [13]. Therefore, many researchers question the possibility of a direct impact by SGLT2is on the heart. A lot of research is underway to explain the beneficial mechanisms of action of this group of drugs on the CV system. Various mechanisms are being discussed, and new aspects of the action of SGLT2is are being discovered. However, many questions remain unanswered. It is fascinating that, for the first time in history, an antidiabetic drug has been shown to reduce CV mortality and the number of hospitalisations due to HF, while the mechanisms of such action are not fully understood. This opens the field for new research and new discoveries, which are important both from the point of view of cardiology and diabetology.

This article presents the current state of knowledge about the mechanisms of action of this extremely interesting group of antidiabetic drugs, which are also currently earning their name as cardiological drugs.

## Diabetes and CV risk

It is well known that type 2 diabetes is associated with a significantly higher risk of CV events such as myocardial infarction and stroke compared to people without diabetes. In turn, CV incidents account for 80% of deaths among patients with type 2 diabetes [1]. The incidence of diabetes is steadily increasing and is now considered a global pandemic. The estimated number of diabetics worldwide is currently 370 million [14]. Diabetes-related hyperglycaemia is a particularly important risk factor for the development of microangiopathic complications such as nephropathy, retinopathy and diabetic neuropathy. The occurrence of microangiopathic complications in diabetes is associated with a high risk of developing macrovascular complications, including atherosclerotic cardiovascular diseases (CVD) [1, 2]. In addition, the clinical characteristics of patients with type 2 diabetes indicate the frequent coexistence of other significant CV risk factors. Over half of patients with type 2 diabetes are obese. According to various sources, hypertension occurs in 20–60% of patients with type 2 diabetes [2]. Diabetic nephropathy, in turn, is the primary cause of chronic kidney disease (CKD), which significantly increases CV risk [15]. To date, interventional studies aimed at reducing hyperglycaemia with the use of, for example, insulin or sulfonylureas, have not significantly reduced CV mortality in patients with type 2 diabetes despite effective glycaemic normalisation [1]. This is generally explained by the common prevalence of moderate to severe insulin resistance, hyperlipidemia, and the aforementioned obesity and hypertension in type 2 diabetes. This combination of CV risk factors is known as metabolic syndrome, which is associated with a significant increase in CV events in type 2 diabetes [1]. This is due mainly to the molecular mechanisms underlying the development of insulin resistance, which are directly involved in the process of atherogenesis. Data confirms that obese people with insulin resistance but without diabetes have an increased CV risk similar to that in patients with type 2 diabetes [1]. This means that in type 2 diabetes, hyperglycaemia is not an independent or major risk factor in the development of atherosclerotic CVD.

## Diabetes and HF

Diabetes is also a key, independent risk factor for HF development, and in patients diagnosed with HF, it is a strong and unfavourable prognostic factor. This applies to both HF with reduced ejection fraction (HFrEF) and HF with preserved ejection fraction (HFpEF) [16–20]. Clinical trials registries show that in patients with type 2 diabetes, HF is almost as common as atherosclerotic CVD. In addition, in this group of patients, HF remains the primary cause of hospitalisation. Diabetes is associated with a higher frequency of asymptomatic left ventricular systolic and diastolic dysfunction. It is also responsible for the more rapid development of symptomatic HF [16]. Although we have data confirming that the control of classic CV risk factors reduces ischaemic events, in the case of HF in patients with diabetes, intensive glycaemic control does not translate into a significant clinical benefit. This indicates that more complex mechanisms underlie the unfavourable prognosis for patients with HF and diabetes. Certainly more factors should be considered in risk stratification in this patient population, and the old glucocentric approach is not entirely appropriate. This is confirmed by interventional studies focused on intensive normalisation of glycaemia in patients with diabetes, in which there was not only no improvement in prognosis in HF but sometimes even an increase in the number of CV incidents with such treatment. Meta-analyses of randomised clinical trials assessing more intensive versus less intensive glucose control in patients with long-term type 2 diabetes showed that the intensive glucose control strategy was associated with a 9% reduction in the relative risk of a complex endpoint, i.e. major adverse cardiovascular events (MACEs). This strategy did not, however, reduce the risk of a fatal/nonfatal stroke, the incidence of peripheral arterial disease, hospitalisation or death due to HF or CV and all-cause mortality [3].

Recent clinical studies assessing a new class of antidiabetic drug, SGLT2is (empagliflozin, canagliflozin and dapagliflozin), showed a significant decrease in both CV and all-cause mortality, as well as a reduction in the number of hospitalisations due to HF in patients with type 2 diabetes treated with these drugs compared to those given a placebo. This effect was achieved regardless of whether CVD or HF existed before the introduction of these drugs. This is undoubtedly a breakthrough in the treatment of this group of patients in relation to the strategies used so far [3, 21–23].

## The target and mechanisms of action of SGLT2is

**Fig. 1:**
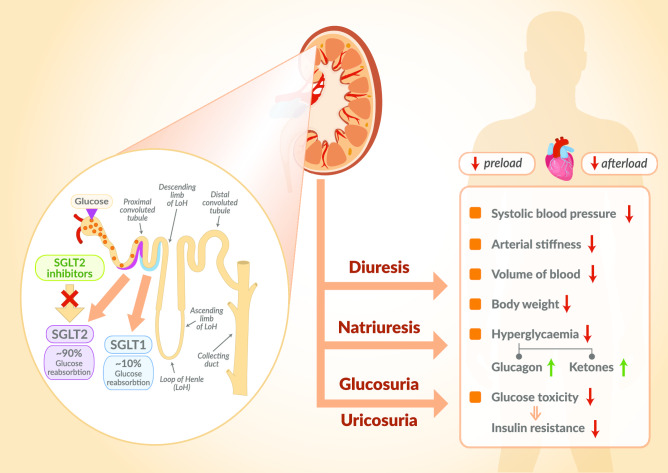
Mechanism of action of SGLT2is. The basic mechanism of action of SGLT2is is to inhibit the active transport of glucose by SGLT2 in the luminal surface of the S1 segment of the proximal renal tubule which is linked to Na^+^ transport maintained by its active extrusion. The latter process takes place with the participation of Na^+^/ATPase of the cell membrane. Under normal circumstances, in the glomeruli of the kidneys, large quantities, about 180 g/day, of glucose are constantly filtering into the primary urine. Then, it is reabsorbed in the proximal tubule with the participation of SGLT2 (90%) and to a lesser extent with the participation of SGLT1 (10%). SGLT2is significantly reduce glucose reabsorption from primary urine at the level of the proximal tubule and thus induce glucosuria, lowering plasma glucose concentration in a mechanism independent of insulin. Simultaneously with glucosuria, SGLT2 inhibitors intensify the excretion of sodium, which is cotransported with glucose and which, in turn, causes an increase in osmotic diuresis. This unique mechanism of SGLT2is action results in numerous non-glycaemic effects throughout the body. SGLT1 sodium-glucose cotransporter-1, SGLT2 sodium-glucose cotransporter-2, SGLT2is sodium-glucose cotransporter-2 inhibitors

The fundamental activity of SGLT2is is to inhibit the active reverse transport of glucose by SGLT2 in the luminal surface of the S1 segment of the proximal renal tubule, which is linked to Na^+^ transport maintained by Na^+^ active extrusion. The latter process takes place with the participation of Na^+^/ATPase of the cell membrane. Under normal circumstances, the renal glomeruli filter about 180 g/day of glucose into the primary urine. Next, it is reabsorbed in the proximal tubule with the participation of SGLT2 (90%) and, to a lesser extent, with the participation of SGLT1 (10%) [3]. SGLT2is significantly reduce glucose reabsorption from the primary urine in the proximal tubule and thus induce glycosuria, lowering plasma glucose in an insulin-independent mechanism. However, the risk of hypoglycaemia is low because these drugs only cause glucosuria when hyperglycaemia occurs. SGLT2 inhibitors simultaneously with glucosuria intensify the excretion of sodium, which is cotransported with glucose. This excretion of sodium, in turn, causes an increase in osmotic diuresis [3] (Fig. 1).

**Fig. 2:**
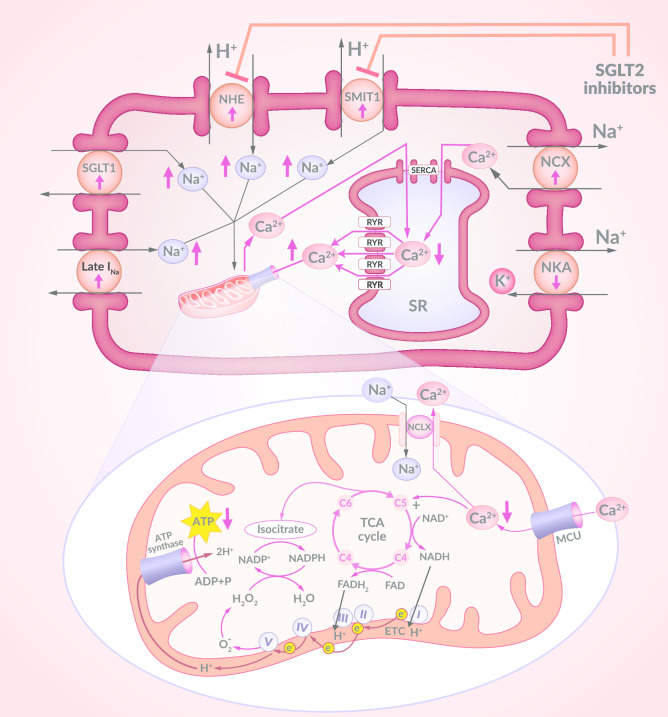
Disruption to the regulation of sodium and calcium concentrations in cardiomyocyte in HF. The intracellular regulation of both Ca^2+^ and Na^+^ concentration levels is disrupted in HF. Ca^2+^ and Na^+^ concentrations in the cardiomyocyte cytoplasm are significantly increased at this time. The transport of Ca^2+^ to SR with the participation of SERCA is interrupted. In addition, the loss of Ca^2+^ from SR by RYR increases. The increase in cytoplasmic Na^+^ concentration in cardiomyocytes in HF occurs, mainly, due to excessive SGLT1 expression, increased Na^+^ influx by Late I_Na_ and increased NHE and SMIT1 activity. The cytoplasmic Na^+^ overload observed in HF causes an increase in Ca^2+^ efflux from the mitochondria to the cytosol, which is mediated by NCLX. The intra-mitochondrial Ca^2+^ concentration is thus reduced, which causes the inhibition of the Ca^2+^ dependent upregulation of dehydgrogenases in the TCA cycle. This leads to a fall in NADH and NADPH production. Reducing the amount of NADH causes a decrease in ATP production and reducing the amount of NADPH results in disruption to the mitochondrial antioxidant defence system. SGLT2is have the ability to inhibit NHE and SMIT1 and thereby reduce the influx of sodium into cardiomyocyte increase during HF, decrease during HF. HF heart failure, SR sarcoplasmic reticulum, SERCA sarco/endoplasmic reticulum Ca^2+^-ATPase, RYR ryanodine receptors, SGLT1 sodium-glucose cotransporter-1, NHE sarcolemmal Na^+^/H^+^ exchanger, SMIT1 sodium–myoinositol cotransporter-1, Late I_Na_ late Na^+^ current, NKA Na^+^/K^+^ ATPase, NCX sarcolemmal Na^+^/Ca^2+^ exchanger, NCLX mitochondrial Na^+^/Ca^2+^ exchanger, MCU mitochondrial Ca^2+^ uniporter, TCA tricarboxylic acid cycle, NAD^+^ nicotinamide adenine dinucleotide (oxidised), NADH nicotinamide adenine dinucleotide (reduced), NADP^+^ nicotinamide adenine dinucleotide phosphate (oxidised), NADPH nicotinamide adenine dinucleotide phosphate (reduced), FAD flavin adenine dinucleotide (oxidised), FADH_2_ flavin adenine dinucleotide (reduced), ETC electron transport chain, e^−^ electron, ATP adenosine triphosphate, ADP adenosine diphosphate, SGLT2is sodium-glucose cotransporter-2 inhibitors

So far, seven SGLT isoforms have been identified. These are SGLT1 to SGLT6 and sodium–myoinositol cotransporter-1 (SMIT1) [13]. Only two of these are found in the hearts of mammals—SGLT1 and SMIT1. The physiological role of SMIT1 in the heart has not yet been clarified. It is known that an overexpression of SMIT1 activates reduced nicotinamide adenine dinucleotide phosphate (NADPH) oxidase 2 (NOX2), increases the production of reactive oxygen species (ROS) and increases glucotoxicity in cardiomyocytes [13]. It has also been confirmed that SMIT1 is closely related to glucose uptake in the heart. The activation of NOX2 by SMIT1 can modulate glucose sensitisation, which in turn can release ion signals (Na^+^ and Ca^2+^) into cells depending on extracellular glucose concentration. The inhibitory concentration (IC_50_) of empagliflozin and cagliflozin for SMIT1 is 8.3 and 5.6 µM, respectively [24, 25]. As for SGLT1, its increased expression and activity in the hearts of patients with diabetes has been confirmed [13]. This is also one of the causes of a significantly increased concentration of sodium ions (Na^+^) in the cytoplasm of cardiomyocytes in a failing heart. Other causes of this sodium overload of cardiomyocytes in an inefficient heart include increased Na^+^ influx through late Na^+^ current (INa) and increased sarcolemmal Na^+^/H^+^ exchanger (NHE) activity. Such an increase in cytoplasmic Na^+^ concentration in cardiomyocytes causes particularly adverse consequences in mitochondria (see below). We have evidence from in vitro studies that SGLT2is have the ability to directly inhibit NHE and thereby reduce the cytoplasmic sodium concentration in cardiomyocytes. They show a strong affinity to the extracellular part of NHE, which has the ability to bind Na^+^ and, thus, they block the increased influx of Na^+^ into the cytoplasm of cardiomyocytes in HF [13, 26]. This target point may be important in understanding the direct effects of SGLT2is on the heart, despite the lack of SGLT2 expression in the human heart (Fig. 2).

## SGLT2 inhibition and clinical outcomes

As mentioned above, the basic evidence for favourable CV outcomes of SGLT2is (empagliflozin, canagliflozin and dapagliflozin) was obtained mainly in three randomised clinical trials: EMPA-REG OUTCOME, CANVAS and DECLARE-TIMI 58 [1–9, 19, 27]. These three clinical trials differed not only in the studied drug but also in relation to the studied patient population [27].

Patients with type 2 diabetes and previously diagnosed CVD took part in the EMPA-REG OUTCOME trial in which empagliflozin was studied. The use of empagliflozin in this group of patients significantly reduced CV mortality without any significant effect on the number of ischaemic events such as non-fatal myocardial infarction or stroke. In addition, empagliflozin significantly reduced all-cause mortality and the number of hospitalisations due to HF [1, 3, 28, 29].

Canagliflozin was evaluated in the CANVAS Program study, which consisted of two clinical trials CANVAS and CANVAS-Renal [3, 20, 28, 30–33]. The whole study involved patients with type 2 diabetes who either had been diagnosed with CVD or simply had risk factors associated with the occurrence of CVD. The use of canagliflozin in this group of patients significantly reduced the occurrence of the composite endpoint such as MACE and significantly reduced the number of HF hospitalisations. Canagliflozin treatment did not, however, reduce the risk of any one single MACE component, i.e. CV mortality, non-fatal myocardial infarction and non-fatal stroke. There was also no reduction in all-cause mortality [3, 20, 34]. This is mainly explained by the fact that a significant part of the entire study population were patients who had no CVD and in whom such favourable clinical effects were not observed. This suggests that the cardioprotective effects of canagliflozin are mainly in the field of secondary prevention [3]. This was confirmed by the Evidence for Cardiovascular Outcomes With Sodium Glucose Cotransporter 2 Inhibitors in the Real World (EASEL) study, which is a retrospective assessment of a large group of patients from the US Department of Defense Military Health System registry [35]. They were patients with type 2 diabetes and confirmed CVD who had begun canagliflozin treatment. The control group consisted of patients from the same registry with similar clinical characteristics who started diabetes treatment with a drug other than SGLT2i. In this group of patients with a high CV risk, assessed in routine clinical practice, the use of canagliflozin was associated with a significantly lower percentage of CV events, CV mortality, and all-cause mortality compared to the patients treated with other antidiabetic medication [35].

The DECLARE-TIMI 58 study examined dapagliflozin in a group of patients with type 2 diabetes with CVD and patients with type 2 diabetes without CVD, but who had numerous atherosclerotic CVD risk factors [3, 19, 36–39]. Dapagliflozin reduced the number of HF hospitalisations and CV mortality in this group of patients. The use of dapagliflozin was associated with 0.8% absolute risk reduction (ARR) and 27% relative risk reduction (RRR) of hospitalisation due to HF. Importantly, this beneficial effect occurred in both patients with known atherosclerotic CV disease and those without it, as well as in patients with a history of HF and those without a history of HF. Further sub-analysis of this study showed that this effect also occurred regardless of whether or not patients had had a myocardial infarction, with ARR greater in patients after myocardial infarction [3, 19, 20, 36–38]. Similar results for dapagliflozin were obtained in a Swedish observational study using Swedish nationwide healthcare registries from 2013 to 2016 [40]. Patients with type 2 diabetes who were starting therapy with dapagliflozin or another antidiabetic drug were selected from this registry. All other inclusion criteria for this observational study were the same as those in the DECLARE-TIMI 58 study. This study, like the DECLARE-TIMI 58 study, confirmed in real life and in routine medical practice that dapagliflozin reduced CV mortality and the number of HF hospitalisations in the study population [40].

Further disparities between these studies may also result from different clinical characteristics of the patient populations studied (in the EMPA-REG OUTCOME trial only patients with confirmed CVD, and in other studies patients both with and without confirmed CVD) [27]. Such a difference explains the period of time taken for divergence of the lines illustrating mortality in the studied groups. In EMPA-REG OUTCOME, this happened very early, in the first 3 months, and in other studies decidedly later. In addition, only empagliflozin reduced both CVD mortality and all-cause mortality. This is explained by the fact that patients in the EMPA-REG OUTCOME study had a higher risk of death at baseline and the management used was secondary prevention. In contrast, in the other two studies, patients without CVD required a longer observation time before the beneficial effects of treatment became apparent [27].

On the other hand, similar results were obtained for all of the three drugs tested in terms of improving renal function in patients with CKD. It is estimated that up to 40% of patients with type 2 diabetes have CKD, which significantly increases morbidity and mortality in this group of patients [3]. Both empagliflozin and canagliflozin like dapagliflozin improve kidney function in patients with diabetes and slow the progression of diabetic nephropathy and albuminuria [30, 31, 33, 34, 41–44]. These beneficial effects of SGLT2is occur both in patients with or without a prior diagnosis of CKD and regardless of whether CVD had been diagnosed or not [42]. Interestingly, the lines showing the differences in kidney function between the actively treated and placebo groups begin to diverge as early as the first month and the beneficial effect is maintained for more than 3 years [33]. All these beneficial effects occur in patients receiving concomitant drugs that inhibit the renin-angiotensin–aldosterone system and who have an estimated glomerular filtration rate (eGFR) above 30 ml/min/1.73 m^2^ of body surface area. These effects were also independent of the effectiveness of glucose lowering during the SGLT2i therapy [3, 41].

The study population and the results of the EMPA-REG OUTCOME study were also used to analyse the cost-effectiveness of empagliflozin therapy plus standard care compared with only standard care [45]. This analysis also used the results of the Liraglutide Effect and Action in Diabetes: Evaluation of Cardiovascular Outcome Results (LEADER) study on liraglutide for the treatment of diabetes. For this analysis, the IQVIA CDM model, which is a Web-based computer simulation model, was used to project the long-term health economic outcomes of the therapies. This model is commonly used in the analysis of the management of diabetes, for a range of different economic analyses, e.g. cost–benefit analysis. This study took into consideration the UK healthcare system perspective and only direct costs were included. It projected the outcomes of the EMPA-REG OUTCOME and LEADER trial in patients with established CVD [45]. The system used in this study was calibrated to project CV and renal outcomes that matched the results of Cardiovascular Outcome Trials (CVOTs). The study demonstrated that empagliflozin plus standard care is a cost-effective treatment compared with only standard care and was considered dominant versus liraglutide plus standard care in the management of patients with both diabetes and confirmed CVD [45]. In other health economic studies based on the EMPA-REG OUTCOME trial, it has also been confirmed that empagliflozin therapy is cost-effective in patients with both diabetes and confirmed CVD [46–48].

The results of the most recent randomised clinical trial Empagliflozin Outcome Trial in Patients with Chronic Heart Failure and a Reduced Ejection Fraction (EMPEROR-Reduced), presented during the European Society of Cardiology (ESC) 2020 Congress and published in the New England Journal of Medicine in August 2020 are of particular interest [49]. This study evaluated 3730 patients, both with and without diabetes, who were suffering from chronic HF with reduced ejection fraction. These patients were given standard HF treatment as per recognised guidelines. The addition of empagliflozin to their treatment plan resulted in a significant 25% RRR in the primary composite endpoint of CV death or hospitalisation due to HF. Furthermore, the addition of empagliflozin to the treatment reduced the incidence of first time and subsequent hospitalisation due to HF by 30% in these patients. This study also confirmed that the use of empagliflozin has a nephroprotective effect. It was shown that in this study population, the use of empagliflozin significantly slows down the decline in glomerular filtration rate and reduces kidney outcomes by 50%. These beneficial clinical effects of empagliflozin administration occurred in both diabetic and non-diabetic patients. Moreover, these benefits were independent of all types of recommended HF treatment, including sacubitril-valsartan. It is also worth emphasising the good safety profile of empagliflozin confirmed in this study. It did not significantly increase the incidence of hypovolemia, hypotension or hypoglycaemic events in either diabetic or non-diabetic patients [49].

The results of randomised clinical trials are confirmed in registry data. A large analysis of six registries from the USA, Sweden, Denmark, UK, Germany and Norway, which included over 300,000 patients with type 2 diabetes, showed significant clinical benefits in terms of CV events with the use of SGLT2is compared to other groups of antidiabetic drugs [50]. In this large real-world study, in which both patients with and those without CV diagnosis participated, it was shown that treatment with SGLT2is versus treatment with another antidiabetic drug resulted in a 39% RRR of hospitalisation due to HF, a 51% reduction in all-cause mortality and a 46% reduction in HF hospitalisation [50]. Interestingly, a large percentage of these patients (87%) had had no prior diagnosis of CVD. The study result was also not affected by the geographical diversity of using different SGLT2is. This supports the class effect of these drugs [50].

## The postulated mechanisms of the beneficial effects of SGLT2is in HF

As previously mentioned, the beneficial clinical effects of SGLT2is, in all likelihood, are not due to an improvement in glycaemic control. In the clinical trials conducted so far, the strategy of intensive glycaemic control with the use of previously used drugs did not translate into a significant reduction in the number of CV incidents, nor a reduction in CV mortality or number of hospitalisations due to HF. Evidence for this has been shown in such large clinical studies as UK Prospective Diabetes Study (UKPDS), Action in Diabetes and Vascular Disease: Preterax and Diamicron MR Controlled Evaluation (ADVANCE) and Action to Control Cardiovascular Risk in Diabetes (ACCORD) [1, 44].

Counter to the concept that the beneficial clinical effects of SGLTis are caused by an improvement in glycaemic control is also the fact that the reduction of haemoglobin A1C (HBA1C) attained by using empagliflozin versus a placebo was small, only 0.45% after 90 weeks and 0.28% after 204 weeks [1]. Moreover, in studies such as UKPDS and Veterans Affairs Diabetes (VADT), achieving the aforementioned 9% RRR of MACE with treatment that improved glycaemic control took a long time—about 10 years. In studies with empagliflozin, a significant reduction in CV mortality was obtained after just 3 months and was still present after 6 months of therapy [1].

Further evidence of the non-glycaemic mechanisms of action of SGLT2is is provided by the Dapagliflozin and Prevention of Adverse-Outcomes in Heart Failure (DAPA-HF) trial, which included 4744 patients with EFrHF who were being optimally treated pharmacologically, in the classic way for HF. Only half of these patients had type 2 diabetes [4, 20, 37, 51–55]. In this study, dapagliflozin or a placebo was added to the standard HF treatment. The use of dapagliflozin in this study was associated with a 26% reduction in the composite endpoint including CV deaths, HF hospitalisation, and emergency visits due to HF exacerbation that required intravenous therapy. In the HF group of patients using dapagliflozin, there was also an 18% reduction in CV mortality and a 17% reduction in all-cause mortality. This effect was not dependent on the age of the patients studied, and this was confirmed by an analysis of the individual endpoints in relation to the age of the patients (patients in this study were aged 22 to 94 years, mean 66.3) [4, 37, 51–54]. This study also investigated the effect of dapagliflozin on the overall health status outcomes using the Kansas City Cardiomyopathy Questionnaire (KCCQ) [53]. The beneficial effect of dapagliflozin in the studied population of HF patients was confirmed in terms of the severity of symptoms, physical activity and quality of life [53]. Importantly, the range of clinical benefits obtained from the use of dapagliflozin was similar in both the group of patients with and those without diabetes. This contradicts the concept that the beneficial effects of SGLTis are only produced by limiting the cardiotoxic effect of hyperglycaemia [4, 20, 37, 51].

In addition, other studies confirming the beneficial effect of SGLT2is on short-term shear stress (SS) and endothelial function in patients with type 2 diabetes have shown that this effect is independent of the level of glycaemic control [56]. SS is the main haemodynamic factor acting on the endothelial surface, stimulating it to produce vasodilators and atheroprotective substances. During reactive hyperaemia arising after transient ischaemia, there is a sharp, short-term increase in SS, which induces vasodilation. This vasodilation is usually measured using the ultrasound-based flow-mediated dilation (FMD) technique. In the study with empagliflozin, it was shown that this drug causes a significant increase in SS in the brachial artery and an increase in FMD of this artery relative to therapy based on incretin drugs. Both treatments resulted in a similar level of glycaemic control. However, only empogliflozin therapy gave beneficial effects in the form of a significant increase in SS and FMD in the brachial artery [56].

What other potential mechanisms of action of this group of drugs may be responsible, therefore, for their beneficial clinical effects? Among others, mechanisms such as an impact on the metabolism of fatty acids and ketone bodies, modulation of mitochondrial function, negative sodium balance and redistribution of sodium ions, reduction in plasma uric acid concentration, an impact on glucagon secretion, weight reduction, and a lowering of blood pressure have all been postulated. In addition, it is believed that through complex regulatory mechanisms associated with some of the processes mentioned above, SGLT2is may also have anti-inflammatory and antioxidant effects. The combined effect of metabolic and haemodynamic factors on the slowing of the atherosclerotic process is also being taken into consideration [1, 12, 13, 26, 57–67].

### Fatty acids and ketone bodies

It has been confirmed that SGLT2is, by inducing glucosuria and hypoglycaemic activity, cause hormonal changes such as a decrease in insulin concentration and an increase in glucagon concentration, which enhances lipolysis in adipose tissue. This promotes the transition of the entire body metabolism from a model based on obtaining energy from glucose to a model based on obtaining energy through oxidation of fatty acids. This has been confirmed for dapagliflozin and for empagliflozin. However, obtaining energy based on fatty acid oxidation is associated with the need for greater oxygen consumption. This is illustrated by adenosine triphosphate (ATP) production/O_2_ consumption ratio (P/O), which is 2.53 for fatty acids and 2.33 for glucose [68]. A change in metabolism towards fatty acid oxidation would therefore be associated with an increase in myocardial oxygen demand and could increase ischaemia in patients with type 2 diabetes. Therefore, such systemic metabolic change, observed in SGLT2is studies, cannot explain the reduction in both CV mortality and the number of hospitalisations due to HF in patients treated with this group of drugs. On the other hand, the intracardiac mechanisms regulating the use of individual substrates for energy production within the myocardium itself in HF may be significant [1].

This is due, inter alia, to the fact that SGLT2is-induced metabolic changes, mainly the reduction of the insulin/glucagon ratio, intensify lipolysis within adipose tissue and fatty acid oxidation in the liver, which enhances ketogenesis. It is these ketone bodies that are considered to be the so-called super fuel that can be used as an additional substrate for energy production in an inefficient heart [60, 68]. Ketone bodies include acetone, acetoacetate (AcAc) and β-hydroxybutyrate (BHB). They are formed mainly in the liver, when there is, among other things, insufficient insulin action or an increased amount of free fatty acids (FFAs) in the course of increased lipolysis. In this situation, the beta-oxidation of fatty acids increases, which leads to an increase in the ratio of reduced nicotinamide adenine dinucleotide (NADH)/nicotinamide adenine dinucleotide (NAD^+^) and, consequently, promotes the conversion of AcAc to BHB in hepatocyte mitochondria. In the process of beta-oxidation of fatty acids, acetyl-CoA, which is the final product of fatty acid oxidation, is formed. Acetyl CoA can either enter the tricarboxylic acid cycle (TCA cycle) or be converted to ketones. It has been shown that SGLT2is, by increasing glucagon secretion, stimulate the latter pathway, thus increasing the number of ketone bodies [1].

Can the heart, however, use ketone bodies effectively to produce energy? The heart’s energy demands are huge, and it has limited storage possibilities. Hence, cardiomyocytes must produce a large amount of ATP. Under normal conditions, the main energy source for cardiomyocytes is FFAs (60%). Carbohydrate oxidation provides 40% of the heart’s energy requirements. In various stress situations, such as hypoxia or pressure overload, these proportions are reversed and carbohydrates become the main source of energy. This is because the glycolysis pathway, as an energy source, can also alternatively operate under anaerobic conditions. However, the efficiency of the glycolysis process in the production of ATP is much weaker than that of mitochondrial oxidative pathways. So, the heart in HF needs more efficient energy sources. In this context, ketone bodies can be a good substrate in the energy production process in HF, because, in this situation, cardiomyocytes are often subject to an insufficient oxygen supply for long periods of time. In such circumstances, a failing heart can more easily use ketone bodies to produce ATP rather than FFAs or glucose. The P/O ratio is 2.50 for ketone bodies and is more favourable than for fatty acids (P/O 2.33). The P/O ratio for glucose is 2.53 and does not differ significantly from the P/O for ketone bodies, but BHB is in a more reduced form than pyruvate and is a more readily available substrate for cardiomyocytes for ATP production. It is also significant that, by mass, the heart is the organ with the highest degree of consumption of ketone bodies [13, 68]. It has also been shown that ketone bodies can increase the efficiency of mitochondrial oxidation at the level of the coenzyme Q complex. An increased expression of genes associated with the oxidation of ketone bodies in cardiomyocytes in HF has also been found at the transcriptional level [13]. We know from other animal studies that ketone bodies can significantly reduce oxidative stress and prolong the life of mice as well as exhibiting anti-inflammatory effects [69, 70]. It has been demonstrated in a mouse model of early diabetes using obese, insulin-resistant homozygous C57BL/6J-lepob (ob/ob^−−^) mice, that SGLT2is have been shown to cause metabolic changes in the form of an increase in plasma glucagon concentration and an increased glucagon/insulin ratio as well as an increase in the concentration of ketone bodies [71]. This experimental model is interesting because these mice typically have, aside from insulin resistance and diabetes, left ventricular systolic dysfunction and microcirculation disorders in the heart. The use of empagliflozin in these mice simultaneously with the metabolic effects described above, including the increased concentration of ketone bodies, resulted in an improvement in left ventricular contractility and an improvement in cardiac microcirculation [71]. Additionally, in another study with diabetic mice, it was confirmed that, compared to the control group, they had a very significant decrease in ATP production in the heart, which was associated with a significant reduction in the level of glucose and fatty acid oxidation and a particularly large reduction in the oxidation of ketone bodies. This eventually led to HF in these animals [72]. The use of empagliflozin in these animals caused, as observed in other studies, an increase in the plasma concentration of ketone bodies and a 31% increase in ATP production in the heart compared to the control group. This increase in ATP production under the influence of empagliflozin was mainly due to an increase in the level of glucose and fatty acid oxidation in the hearts of these mice and not due to the direct effect of empagliflozin on the oxidation of ketone bodies. However, it is believed that the increase in plasma ketone body concentrations observed in these mice under the influence of empagliflozin is an additional source of substrates for ATP production, regardless of the level of glucose or fatty acid oxidation [72]. This concept is supported by data from the same study, in which, additionally, BHB was used as an intravenous infusion in diabetic mice with reduced levels of ATP production, leading to a significant increase in ATP production in the heart. This did not occur through changes in the level of glucose or fatty acid oxidation, but only through an independent, significant increase in the oxidation of ketone bodies. In addition, when the hearts from these diabetic mice were collected and ex vivo given a perfusion of nutrient fluid with a high concentration of ketone bodies, such as was found in the plasma of the mice treated with empagliflozin, a significant increase in ATP production was obtained in these hearts. This suggests that the beneficial effects of empagliflozin in diabetes and HF are associated with its impact on increased ATP production in the heart [72]. Similar results were obtained in doxorubicin-induced HF mice in which the use of empagliflozin significantly increased BHB plasma concentrations [73]. This experimental model is a recognised animal model of non-diabetic HF. Empagliflozin has been shown to inhibit the development in these mice of doxorubicin-induced cardiomyopathy, which is associated with an increase in the plasma concentration of ketone bodies. This study confirmed that BHB improves cell survival and reduces ROS production in mice treated with doxorubicin. In addition, BHB increased ATP production and restored impaired mitochondrial function in cardiomyocytes [73].

Hence, increasing the level of ketone bodies with SGLT2is may have beneficial effects in patients with HF.

### The modulation of mitochondrial functions

Mitochondria fulfil some particularly important functions in cardiomyocytes, which include not only energy production but also metabolic functions related to intracellular signal transduction and regulation of apoptosis. Mitochondria have the ability to fuse together and undergo fission. This is regulated by complex mechanisms and allows the mitochondrial functions to be tailored to the prevailing needs. They can significantly increase their energy production capacity by fusing together intensively. This happens in certain situations related to excessive stress on the heart. In this situation, an increased fusion of mitochondria occurs, which results in an increase in ATP production and allows cardiomyocytes to survive the stressful situation. At other times, when damaged mitochondria have to be removed from the cell, they undergo a process of division into smaller parts and mitochondrial autophagy, which is called mitophagy [74]. Both of these processes are strictly controlled. Mitochondrial fusion is regulated by mitofusion1 (Mfn1), mitofusion2 (Mfn2) and Opa1. In contrast, mitochondrial fission is regulated by Dynamin-related protein 1 (Drp1) and mitochondrial fission factor (Mff). There is evidence that mitochondrial fusion disorder, through the inhibition of Mfn1 and Mfn2, results in increased cardiac contraction dysfunction in both basal and stressful conditions [75]. In addition, mitochondrial fission disorder by pharmacological inhibition of Drp1 reduces the extent of myocardial damage as a result of experimentally induced ischaemia/reperfusion [76].

From animal studies, there is evidence that SGLT2is can have a positive effect on the dynamics of the mitochondrial fusion/fission changes and thus modulate the mitochondrial function. In an animal model using rats, in which mitochondrial dysfunction was induced by a high fat diet, the use of ipragliflozin improved mitochondrial function by restoring the appropriate levels of Mfn2 and Opa1 [77]. Moreover, it has been demonstrated in the rat model of metabolic syndrome that the use of dapagliflozin normalises the Mfn1/Mfn2 ratio. This leads to a suppression in the prolongation of ventricular repolarisation process in these animals [78]. In turn, empagliflozin normalises the adenosine monophosphate (AMP)/ATP ratio and thus activates adenosine monophosphate-activated protein kinase (AMPK). Activation of AMPK modulates Drp1 phosphorylation in such a way that mitochondrial fission is inhibited in cardiomyocytes [79]. Empagliflozin has also been shown to normalise the size and number of mitochondria in cardiomyocytes of diabetic rats. Empagliflozin also inhibited diabetes-induced reduction in mitochondrial size in cardiomyocytes of rats with experimentally induced myocardial infarction. This occurred because empagliflozin inhibited the excessive activity of mitochondrial fission 1 protein (Fis1) and the resulting excessive production of ROS. The end result, observed in this experimental model, was that the size of the myocardial infarction was reduced by empagliflozin [80].

These studies confirmed that SGLT2is modulate both the fusion and fission processes and can thus positively affect mitochondrial function in cardiomyocytes in HF. However, the molecular mechanisms of this modulation remain unexplained. It is also unknown whether these examples of the effects of particular SGLT2is can be considered a class effect or whether they are specific only to a particular drug. A separate issue, requiring further research, is the precise determination of the contribution of fusion and fission processes in HF development.

### Na^+^ redistribution in cardiomyocytes

Since SGLT2 transports both glucose and sodium in the proximal tubule of the nephron, SGLT2is also induce natriuresis in parallel to glucosuria. The inhibition of SGLT2 causes a negative sodium balance, with no change in plasma sodium concentration. However, sodium is redistributed between the extracellular and intracellular compartments [12, 13, 68]. Intracellular sodium homeostasis is changed. Sodium plays an important role in the mitochondrial oxidation–reduction processes and in the regulation of electromechanical coupling in cardiomyocytes. The regulation of Na^+^ concentration in cardiomyocytes is closely related to the regulation of Ca^2+^ concentration and is dependent on the activity of sarcolemmal Na^+^/Ca^2+^ exchanger (NCX) and mitochondrial Na^+^/Ca^2+^ exchanger (NCLX). Physiologically, NCX in cardiomyocytes mainly removes calcium from the cell to the extracellular space. However, in the early phase of action potential, depending on the transmembrane concentration gradient of Na^+^ and Ca^2+^, it launches Ca^2+^ transport into the cytosol. In contrast, NCLX in cardiomyocytes is mainly responsible for the release of calcium from the mitochondria into the cytosol. In HF, the intracellular regulation of both Ca^2+^ and Na^+^ levels is disturbed. Ca^2+^ and Na^+^ levels in the cardiomyocyte cytoplasm are significantly increased in HF [13, 68]. Ca^2+^ transport into the sarcoplasmic reticulum (SR) involving sarco/endoplasmic reticulum Ca^2+^-ATPase (SERCA) is disrupted. In addition, the loss of Ca^2+^ from SR by ryanodine receptors increases [13]. The increase in cytoplasmic Na^+^ concentration in cardiomyocytes in HF occurs mainly due to excessive SGLT1 expression and increased Na^+^ influx by late I_Na_ and increased NHE activity [12, 13, 26, 68] (Fig. 2).

The Na^+^ cytoplasm overload, observed in HF, causes an increase in Ca^2+^ efflux from the mitochondria to the cytosol. This efflux occurs thanks to NCLX. As a result of this process, the intra-mitochondrial Ca^2+^ concentration is reduced, which suppresses the Ca^2+^ dependent upregulation of dehydgrogenases in the TCA cycle. This leads to a decrease in NADH and NADPH production. Reducing the amount of NADH causes a decrease in ATP production, while reducing the amount of NADPH results in a disruption in the mitochondrial antioxidant defences, on account of NADPH being the electron donor for such antioxidant enzymes as peroxiredoxins, glutathione peroxidase and glutaredoxin. When there is an excess of cytoplasmic Na^+^ in mitochondria, they become a source of ROS. In this way, a Na^+^ overload of the cardiomyocyte cytoplasm results in increased oxidative stress, which enhances neurohumoral activation in HF and has a pro-arrhythmic effect [13, 68]. Reducing the Na^+^ overload of cardiomyocite cytoplasm and, thus, achieving an improvement in mitochondrial energy production and antioxidant defences, is becoming an attractive therapeutic strategy in HF. Recent studies indicate that SGLT2is may have a positive effect on the cytosolic concentrations of Na^+^ in cardiomyocytes despite the lack of SGLT2 expression in the human heart under both normal and HF conditions. It has been confirmed in in vitro studies that empagliflozin, canagliflozin and dapagliflozin all reduce the cytosolic concentrations of Na^+^ by directly inhibiting NHE and SMIT1 flux [13, 68] (Fig. 2).

### Reduction of plasma uric acid concentration

It is also sometimes hypothesised that SGLT2is, by enhancing renal excretion of uric acid and generating a moderate reduction of plasma uric acid, may have a positive effect on CV risk. However, the observed effect of SGLT2is on plasma uric acid concentration was small with a reduction of about 0.7 mg/dl [1]. In addition, although it has been observed that the occurrence of hyperuricemia is associated with a higher CV risk, the causal relationship remains controversial. In interventional studies, aimed at reducing uric acid concentration, no benefit in reducing CV risk has been demonstrated. Therefore, it is considered unlikely that the reduction in CV mortality, obtained in clinical trials with SGLT2is, can be explained by a decrease in plasma uric acid levels. There is agreement, however, that a decrease in plasma uric acid may play a part in the significant slowdown in the development of diabetic nephropathy as confirmed in the EMPA-REG OUTCOME study [1].

### The effect on glucagon secretion

As it has been confirmed that SGLT2 is expressed in alpha cells in the pancreas and plays an important role in regulating glucagon secretion, the effect of SGLT2is on glucagon secretion was also investigated. Both empagliflozin and dapagliflozin have been shown to cause a significant increase in plasma glucagon levels. Studies on the effects of glucagon on the heart have shown that intravenous glucagon infusions in humans do not affect left ventricular function [1]. It is therefore unlikely that an increase in plasma glucagon concentrations directly contributes to a reduction in CV mortality and a reduction in HF hospitalisation in patients treated with SGLT2is.

### Reduction in body mass

The inhibition of SGLT2 by SGLT2is in the proximal tubule of the nephron results in glucosuria. This, in turn, is associated with calorie loss and promotes a reduction in body mass. Indeed, in clinical trials with SGLT2is, these drugs have been shown to reduce body weight. For example, in the EMPA-REG OUTCOME study, those people treated with empagliflozin achieved an average weight reduction of approximately 2 kg [1]. Canagliflozin has also been shown to reduce body weight. Reduction of both subcutaneous and visceral adipose tissue was confirmed in people treated with canagliflozin. Additionally, patients treated with canagliflozin had a significantly greater reduction in visceral fat than subcutaneous fat [44]. Although this is obviously a beneficial clinical effect and may have an impact on CV risk, it does not, however, explain such an early significant reduction in CV mortality, occurring after only the first 3 months of SGLT2is treatment [1, 12].

### Reduction in blood pressure

The use of SGLT2is promotes glucosuria and natriuresis and intensifies osmotic diuresis (Fig. 1). Therefore, this group of drugs may have the ability to lower blood pressure. Hypertension is an important risk factor for CV. A reduction in blood pressure by SGLT2is could therefore explain the significant reduction in CV mortality and the number of HF hospitalisations seen in clinical trials using this group of drugs. Indeed, in the EMPA-REG OUTCOME study, most patients had hypertension, but more than 90% of them used antihypertensive drugs, and their blood pressure was well controlled. The mean reduction in blood pressure obtained with empagliflozin in this study was 5/2 mmHg and was maintained for over 3 years. Theoretically, such a reduction in blood pressure may translate into a CV outcome, but in studies on antihypertensive drugs, a significant reduction in CV incidents occurred after 1 year of use, and not, as in the EMPA-REG OUTCOME study, after 3 months. In addition, in clinical trials for the treatment of hypertension, the reduction in blood pressure was associated with a significant reduction in the incidence of strokes and this effect was usually greater than the reduction in cardiac incidents alone. In the EMPA-REG OUTCOME study, the use of empagliflozin was associated with a small, statistically insignificant increase in the number of strokes in the studied group of patients. Researchers conclude, therefore, that it is difficult to account for such a large and rapid decrease in CV mortality in clinical trials using SGLT2is, purely on the basis of this reduction in blood pressure alone. The possibility has been discussed that the brachial arterial blood pressure assessed in patients during SGLT2is studies does not reflect the central aortic blood pressure. This central aortic pressure could have been higher in these patients than the brachial artery pressure. There is no such available data. Additionally, there is no data on aortic stiffness in these patients. It is known from other studies that both elevated central aortic pressure and aortic stiffness are independent indicators of the risk of CV incidents. It has been shown that regardless of the value of arterial pressure measured on the brachial artery, a reduction in the central arterial pressure is associated with a significant reduction in CV events and it is believed that this may have a greater impact on the reduction of cardiological events and HF than on the number of strokes [1]. Although there is no clinical trial data on the effects of SGLT2is on central arterial pressure, the mechanism of action of these drugs by inducing osmotic diuresis and thus reducing intravascular volume may suggest that they have a greater effect on central arterial pressure than on arterial pressure measured on the brachial artery. The reduction in central arterial pressure in the aorta together with the reduction in aortic stiffness results in a reduction in left ventricular afterload, which can reduce its workload, reduce oxygen demand and improve left ventricular function. There is data from animal studies which confirms that the use of empagliflozin significantly reduces aortic stiffness [81]. This has been shown in diabetic mice with a diabetes-induced increase in aortic stiffness. This was confirmed in these mice both in vivo by measuring pulse wave velocity (PWV) and ex vivo by measuring endothelial cell (EC) stiffness, using a special research technique called atomic force microscopy (AFM) [81]. Similar results were obtained in another study in which diabetic mice were given dapagliflozin [82]. In this study, measurements of aortic stiffness were performed in vivo with PWV, providing evidence that dapagliflozin significantly reduces aortic stiffness in diabetic mice [82]. The use of empagliflozin alone and in combination with metformin also significantly reduced arterial stiffness relative to metformin alone in patients with type 1 diabetes [83]. Arterial stiffness measurements were made using PWV as carotid PWV (cPWV) and carotid-femoral PWV (cfPWV) and by common carotid artery stiffness (beta-stiffness). Within all these parameters, a significant improvement was obtained as a result of the use of empagliflozin over metformin [83]. Together with the results of the abovementioned animal studies, this could explain the beneficial impact of empagliflozin on cardiological events, which is particularly strong in patients with coronary artery disease and HF, without significantly affecting the number of strokes. However, there is no data from the EMPA-REG OUTCOME study regarding left ventricular function in the patients studied, both at baseline and during treatment. More research is needed to assess the effect of SGLT2is on central arterial pressure, aortic stiffness and left ventricular function. What is known from the EMPA-REG OUTCOME study is that the favourable CV outcomes obtained were not dependent on the dosage of empagliflozin administered. The mechanism of action of empagliflozin responsible for the hypotensive effect in the form of glucosuria and natriiuresis and osmotic diuresis was at its maximum at a dose of 10 mg per day. There were no differences between the 10 mg and 25 mg daily doses in this range. This could explain the beneficial effect of empagliflozin on central arterial pressure and thus on the CV outcome.

Discussions are still ongoing over the 35% reduction in hospitalisation due to HF achieved in the EMPA-REG OUTCOME study and the short time in which it was achieved. Initially, it was thought that this significant reduction in the number of hospitalisations due to HF and the reduction in CV mortality resulted mainly from the effects of empagliflozin achieved in a group of patients with impaired left ventricular systolic function and HF. Further sub-analysis of this study showed that this reduction is similar in the group of patients who had a diagnosis of HF at baseline and in the group without such a diagnosis. However, it should be remembered that patients in this study were qualified to the appropriate group based on an interview without an echocardiographic assessment of left ventricular function. In addition, patients, who did not present with HF in the interview, but developed HF during the study, stayed in the non-HF group because the information from the interview when the study began still applied. In the absence of any data on left ventricular function in the patients in this study, it should be assumed that many patients, who did not report a history of HF at the beginning of the study, actually had HF at baseline and were incorrectly classified. For this reason, many researchers still believe that the main benefits from the use of empagliflozin were derived by those patients with HF. This also accounts for the rapid effect of empagliflozin in the form of a reduction in CV mortality and a reduction in hospitalisation due to HF. It is believed that this can be explained by the unique, simultaneous reduction of preload, due to a reduction in intravascular volume, and a decrease in the afterload by reducing arterial pressure (mainly central) and aortic stiffness. These two things together could be particularly beneficial in patients with impaired left ventricular systolic function and HF [1, 84]. Although the reduction in intravascular volume by empagliflozin could lead, additionally, to an activation of the renin-angiotensin-aldosterone system, and thus, from the point of view of a CV prognosis, to the unfavourable activation of the AT1 receptor for angiotensin II, this possibility is excluded due to widespread use in this group of patients of angiotensin-converting-enzyme inhibitors (ACEis) or angiotensin II receptor blockers (ARB). On the contrary, this could favour the activation of the AT2 receptor for angiotensin II, which may result in beneficial effects such as vasodilator, antiproliferative, anti-arrhythmic or anti-inflammatory effects [1].

Further studies of SGLT2is which will precisely measure central arterial pressure, aortic stiffness and left ventricular function both at baseline and during treatment are needed. These studies, in addition, should assess brain natriuretic peptide (BNP) and N-terminal pro-hormone of brain natriuretic peptide (NT-proBNP). These measurements will probably provide valuable data to better understand the mechanisms of action of SGLT2is and to identify subgroups of patients who would benefit most from therapy with these drugs.

### The combined effect of metabolic and haemodynamic factors on the slowing of atherosclerosis

Bearing in mind the beneficial effects of SGLT2is mentioned above, in terms of weight reduction, blood pressure and a slight increase in the concentration of high-density lipoprotein (HDL) cholesterol, the multidirectional effect of this group of drugs on the slowing of atherosclerosis should also be considered. However, despite the significant reduction in CV mortality obtained in SGLT2is clinical trials, there was no benefit from these drugs in non-fatal atherosclerotic CV incidents, such as myocardial infarction or stroke. It is believed that this may primarily be due to the duration of the study, which could have been too short to demonstrate a reduction in atherosclerotic CV events. In addition, plasma concentrations of low-density lipoprotein (LDL) cholesterol, which is a very potent atherogenic factor, which significantly increases CV risk, was slightly raised in patients treated with empagliflozin in the EMPA-REG OUTCOME study. This, therefore, could even have an adverse effect on the atherogenesis process in patients treated with empagliflozin [1]. Hence, data, so far, has been unable to prove that the beneficial effects of SGLT2is are caused by their influence on slowing down the atherosclerotic process.

### The anti-inflammatory and antioxidant effects of SGLT2is

As mentioned above, diabetes is a key and independent risk factor for HF development, and, in patients diagnosed with HF, it is a strong, unfavourable prognostic factor [16–20]. HF remains the primary cause of hospitalisation for patients with diabetes. Diabetes is associated with a higher incidence of asymptomatic left ventricular systolic and diastolic dysfunction. It is also responsible for the more rapid development of clinically apparent HF [16]. Progressive fibrosis and cardiac remodelling are the fundamental processes connected with structural and functional abnormalities. There are, therefore, many studies investigating the processes which activate these disorders. The processes which significantly stimulate heart fibrosis and its remodelling and thus contribute to HF progression are inflammation, activation of the immune system and oxidative stress. From experimental evidence currently available, it appears that both inflammation and oxidative stress play a role in the development and course of HF of various aetiologies [85–89]. In patients with diabetes, HFpEF is particularly common. This has a special, metabolic, pro-inflammatory status, which is accompanied by disturbances in the cardiac microvascular endothelial cells’ (CMEC) function. In turn, this is associated with the impaired production and/or availability of nitric oxide (NO), increased ROS production and activation of the inflammatory process [90]. Many inflammatory biomarkers have also been studied, in order to appraise their usefulness as diagnostic and prognostic indicators in HF [85, 88, 89].

One of the distinctive markers of inflammation is C-reactive protein (CRP). Patients with HF have a higher plasma concentration of CRP and, for these patients, this is regarded as an independent predictor of future unfavourable events [91–96]. Its usefulness as a prognostic indicator in HF has been studied particularly often in patients with HFpEF [97]. CRP plasma levels correlated significantly with clinical and laboratory HF severity markers such as New York Heart Association (NYHA) and NT-proBNP. Furthermore, it emerged that CRP is both a powerful and independent predictor of all-cause mortality and is an especially strong predictor of CV mortality [97].

Additionally, the plasma concentration of tumour necrosis factor-alpha (TNF-alpha) correlates well with the NYHA functional class in patients with HF and with the concentrations of classic HF biomarkers, such as NT-proBNP [91, 98, 99]. In animals, who were studied during an experimental myocardial infarction, the participation of CD4+ T lymphocytes in repair processes and positive left ventricular remodelling after myocardial infarction was confirmed [100]. Clinical studies established a correlation between circulating inflammatory cells and biomarkers of inflammation and the degree of left ventricular dysfunction in patients with HF [101]. Elevated plasma concentrations of such biomarkers as TNF-alpha, interleukin-1 (IL-1), interleukin-6 (IL-6), interleukin-8 (IL-8), galectin-3 or growth differentiation factor 15 (GDF15) are widely believed to be typical in HF [85, 101–103].

So far, various anti-inflammatory therapeutic strategies in HF have been evaluated, which, unfortunately, have often not met expectations [85, 103–105]. Studies evaluating the antioxidant effects of various therapeutic strategies are relatively few [64]. In addition to other effects, studies examining SGLT2is also found anti-inflammatory effects in this group of drugs. It has been confirmed that SGLT2is can reduce the plasma concentration of TNF-alpha and IL-6. In this class of drugs, empagliflozin reduces plasma concentrations of pro-inflammatory cytokines more effectively than canagliflozin [61]. In the context of the abovementioned pathogenetic relationships between pro-inflammatory cytokines and the development and course of HF, this could explain the favourable CV outcomes of SGLT2is, particularly in reducing hospitalisation due to HF. One of the concepts is based on the results of research in cell culture conditions in which the normal CMEC function has been shown to determine the correct systolic and diastolic function of cardiomyocytes [90]. When NO synthase was blocked in CMEC with L-NG-nitroarginine methyl ester (L-NAME), CMEC did not have a beneficial effect on cardiomyocytes cultured with them, as previously observed with normal NO production by CMEC. These studies showed that CMEC exposure to TNF-alpha significantly reduces NO bioavailability and thereby cancels out the beneficial effects of CMEC on the systolic and diastolic function of cardiomyocytes. Empagliflozin has been found to significantly reduce this adverse effect of TNF-alpha on CMEC and NO bioavailability, and thereby improve cardiomyocyte function. However, this is not a direct effect on cardiomyocytes or an increase in NO production in CMEC. The effect of empagliflozin on the nuclear factor-kappaB (NF-kappaB) pathway, which is strongly activated by TNF-alpha, was also ruled out. Empagliflozin has been shown to increase NO bioavailability by powerfully weakening ROS production which is induced by TNF-alpha [90]. As ROS can effectively eliminate NO, empagliflozin, by reducing the amount of ROS, can automatically increase the bioavailability of NO. However, in vitro studies have not confirmed any direct antioxidant activity of empagliflozin. It also does not affect NOX4, which is important in the TNF-alpha induced production of ROS. Empagliflozin’s effect on the expression of such ROS-scavenging enzymes as superoxide dismutase 1 (SOD1) and superoxide dismutase 2 (SOD2) under conditions of stimulation by TNF-alpha has also not been confirmed [90]. The only mechanism of empagliflozin’s antioxidant activity confirmed in this study is the very strong inhibition of mitochondrial ROS production in CMEC, which is stimulated by TNF-alpha. Empagliflozin, therefore, by initiating intracellular mechanisms, eradicates TNF-alpha-induced overproduction of ROS in CMEC. This leads to a significant reduction in the amount of ROS in the CMEC cytoplasm and an increase in NO bioavailability, which has been shown to significantly improve the systolic and diastolic function of cardiomyocytes [90].

These interrelated, inflammatory and free radical mechanisms that are directly related to the vascular endothelial function have also been studied in an animal model of diabetes using rats with streptozotocin (STZ)-induced type 1 diabetes [65]. These rats with STZ-induced diabetes were typically found to have increased TNF-alpha and interferon-gamma (INF-gamma) expression as well as increased ROS production. The activation of NOX1 and NOX2 as sources of ROS, disruption of the bioavailability of NO and its signals transmitted through cGMP, as well as persistent low-grade inflammation were all confirmed. These studies have shown that all these adverse phenomena are normalised under the influence of empagliflozin, which translates into improved vascular endothelial function. Improvement in endothelial function was confirmed in an acetylcholine test by studying the relaxation in rat aortic rings under organ bath conditions [65]. These results were confirmed by similar studies on aortic rats with induced diabetes, in which empagliflozin was also evaluated [63]. The mechanisms of all these effects of empagliflozin are complex and include, in addition to the hypoglycaemic effect of this drug itself, the impact on intracellular calcium ion concentrations, which are associated with intracellular sodium ion concentrations. Intracellular calcium concentrations are important due to the interaction of mitochondrial ROS production and NOX activation in the generation of oxidative stress. In this animal model of diabetes, it has also been confirmed that in the course of diabetes, an important, mitochondrial antioxidant enzyme, aldehyde dehydrogenase (ALDH-2) is inactivated. This enzyme degrades toxic aldehydes such as acetaldehyde, malondialdehyde and 4-hydroxynonenal. We have experimental data confirming the important role of ALDH-2 in reducing the area of myocardial infarction during induced ischaemia/reperfusion along with data confirming its cardio-protective effect during experimentally induced myocardial infarction in diabetes [63]. This is why the action of empagliflozin, which improves the activity of this antioxidant enzyme, is so important. This was demonstrated, with a high dose of this drug, in the study cited above [63, 65].

Furthermore, in a mouse model, it was confirmed that in genetically determined type 2 diabetes mice (KK-Ay mice), there was an elevation in ROS production and the associated lipid peroxidation in the heart as well as an increase in myocardial fibrosis [64]. In this particular study, oxidative stress parameters such as lipid hydroperoxides, glutathione peroxidase (GSH-Px), SOD and malondialdehyde (MDA) were assessed directly in the hearts of the animals tested. In the group of diabetic animals, lipid hydroperoxides and MDA levels were significantly higher, while GSH-Px and SOD were significantly lower compared to the control group. This group of diabetic animals also had an increased expression of NOX4, which is the primary source of ROS in these animals. The use of empagliflozin in diabetic mice normalised all of these oxidative stress parameters and significantly reduced the increased NOX4 expression in diabetes. The results of this study confirm that empagliflozin can significantly reduce increased oxidative stress by intensifying the expression of antioxidant enzymes and reducing ROS production, thus limiting the adverse effects of oxidative stress. It was also confirmed that empagliflozin inhibits oxidative stress in the heart of the animals studied by activating the nuclear factor erythroid 2 (Nrf2)/antioxidant response element (ARE) pathway, which is the primary signal pathway involved in the regulation of oxidative stress. It was confirmed in this study that empagliflozin intensifies the translocation of Nrf2 into the cell nucleus, activating the Nrf2/ARE signalling pathway and thus reducing oxidative stress in diabetic mice [64]. One of the further beneficial effects of empagliflozin, demonstrated in this study, is a significant reduction in the expression of transforming growth factor-Beta1 (TGF-Beta1), whose expression is significantly increased in the myocardium of the tested animals with diabetes. TGF-Beta1 is a known mediator of the fibrotic process in the organs and regulates fibroblast function [106]. TGF-Beta1 also regulates the severity of myocardial inflammation during HF. TGF-Beta1 is a cytokine with a multidirectional effect that regulates processes such as the proliferation, differentiation or apoptosis of cells via autocrine and paracrine signalling pathways and through various receptors on the cell surface. TGF-Beta1 also acts as a regulator of both extracellular matrix synthesis and the repair processes for tissue after it has been damaged [103, 107–110]. The abovementioned actions of TGF-Beta1 occur mainly through the TGF-Beta1/mothers against decapentaplegic homolog 3 (TGF-Beta1/Smad3) signalling pathway. The phosphorylation and activation of Smad proteins, which have the ability to bind to specific deoxyribonucleic acid (DNA) and act as transcription factors, is the outcome of the combination of TGF-Beta1 with its TBetaRI and TBetaRII receptors. This process has an impact on various cytokines, such as platelet-derived growth factor (PDGF), fibroblast growth factor (FGF) and TNF. When the TGF-Beta1/Smad3 signalling pathway is impeded, there has been shown to be a reduction in collagen synthesis in cardiac fibroblasts, a reduction in myocardial fibrosis and the prevention of adverse remodelling in cases of pathological overload or damage to the left ventricle [103, 111–114]. It has been confirmed in the HF animal model that the severity of myocardial inflammation is significantly reduced by inhibiting the TGF-Beta1/Smad3 signalling pathway [114]. In the abovementioned study using a mouse model of diabetes, a high level of activation of the TGF-Beta1/Smad3 pathway with high levels of TGF-Beta1 expression and Smad1, Smad2, Smad3 proteins as well as high levels of type I and III collagen in the myocardium of the diabetic mice were confirmed [64]. With the use of emapagliflozin in these animals, all these aforementioned parameters were significantly reduced in the myocardium of these diabetic mice. In addition, empagliflozin has been shown to significantly increase the expression of SMAD7, a known TGF-Beta1 and myocardial fibrosis inhibitor in these animals [64].

Thus, empagliflozin reduces the severity of oxidative stress by activation of the Nrf2/ARE signalling pathway and reduces myocardial fibrosis by inhibiting the TGF-Beta1/Smad3 signalling pathway.

Future research devoted to the specific mechanisms of the anti-inflammatory and antioxidant activity of SGLT2is is, however, necessary to explain how the anti-inflammatory and antioxidant activity of this group of drugs translates into such a significant and early reduction in CV mortality and a reduction in hospitalisation due to HF in patients with diabetes.

## The importance of SGLT2is as cardiological drugs now and in the future

SGLT2is were produced for the treatment of patients with type 2 diabetes. Thanks to their unique mechanism of action, which consists of selective inhibition of the sodium-glucose cotransporter in the proximal tubule of the nephron, they have been, from the beginning, a valuable group of antidiabetic drugs. By increasing urinary glucose excretion, they act as hypoglycaemic agents in an insulin-independent mechanism. SGLT2is, simultaneously with glucosuria, increase sodium excretion, which in turn causes an increase in osmotic diuresis [3]. However, the large clinical trials cited at the beginning of this article showed that this group of drugs also exhibits effects that go beyond the expected hypoglycaemic effects. SGLT2is have been shown to significantly reduce CV mortality, the number of hospitalisations due to HF and all-cause mortality. The beneficial effects of this new group of antidiabetic drugs were totally unexpected. They could not be explained by the hypoglycaemic effects of these drugs alone. The clinical trial results obtained have encouraged numerous studies aimed at explaining the mechanisms of action of the SGLT2is drugs. In addition, the results of these clinical trials transpired to be so significant that they influenced the latest guidelines on diabetes and pre-diabetes associated with CVD issued by the European Society of Cardiology and developed in cooperation with the European Society for the Study of Diabetes [115]. In order to prevent CV incidents, these guidelines recommend the inclusion of a SGLT2is drug in patients commencing treatment for type 2 diabetes if a CVD with atherosclerosis exists or if the patient has a high/very high risk of CVD. It is worth emphasising that this is a class I recommendation with the highest (A) level of evidence. The same applies to patients in this group who have been treated up until now with metformin. They also should have empagliflozin, canagliflozin or dapagliflozin incorporated in their treatment in order to prevent CV incidents. This fact alone demonstrates the importance of this group of drugs. For the first time in history, based on this strong evidence from randomised clinical trials, guidelines recommend the use of a specific group of drugs in a specific group of patients with type 2 diabetes to prevent CV incidents. Thus, SGLT2is has already significantly changed our clinical practice, creating a completely new standard in the management of patients with diabetes.

Considering the increasing number of basic studies and clinical trial data, further changes may be expected in the management of treatment for these patients. More and more scientific data indicates that the favourable CV outcomes of this group of drugs in clinical trials were mainly due to the non-glycaemic effects of their action. The slight reduction in HBA1C achieved by using SGLT2is versus placebo in clinical trials supports this. In addition, we know from previous research on hypoglycaemic treatment in diabetes that even if an RRR of CV events was achieved, it would take years. In studies with empagliflozin, a significant reduction in CV mortality was obtained after just 3 months of therapy. Strong evidence for the non-glycaemic mechanisms of SGLT2i activity is provided by the DAPA-HF study described above. Only half of the patients enrolled in this study had type 2 diabetes. The use of dapagliflozin in this study was associated with a 26% reduction in the composite endpoint including CV death, HF hospitalisation or emergency hospital visits due to HF exacerbation that required intravenous therapy. In the HF group of patients using dapagliflozin, there was also an 18% reduction in CV mortality and a 17% reduction in all-cause mortality. The clinical benefits obtained were similar in both diabetic and non-diabetic patients. This gives a completely new perspective on the use of these drugs in patients with HF, especially since we have more and more scientific research explaining the new mechanisms of action for this group of drugs. Many of these mechanisms can be very beneficial in HF. These include, as described above, the modulation of fatty acid metabolism, the greater use of ketone bodies as substrates for the production of ATP in cardiomyocytes in HF and the beneficial effect of SGLT2is on the function of cardiomyocyte mitochondria. These may be potential targets for these drugs in HF, and not only for diabetic patients.

Moreover, the effect of SGLT2is on the redistribution of sodium and calcium ions, described above, appears to have significant practical implications. SGLT2i mechanism of action is also associated with additional antioxidant effects. Where there is an Na^+^ cytoplasmic excess, mitochondria become a source of ROS and oxidative stress intensifies [13, 68]. Reducing the Na^+^ overload of cardiomyocyte cytoplasm results in improved mitochondrial energy production and antioxidant defence. Using this mechanism of action of SGLT2is is becoming an attractive, potential therapeutic strategy in HF, which, in the future, may be useful not only for patients with diabetes, but others too. Recent studies indicate that SGLT2is may have a positive effect on the cytosolic Na^+^ concentration in cardiomyocytes despite the lack of SGLT2 expression in the human heart under both normal and HF conditions. It has been confirmed in in vitro studies that empagliflozin, canagliflozin and dapagliflozin all reduce the cytosolic Na^+^ concentration by directly inhibiting NHE flux [13, 68].

Equally interesting is the possible future use, in HF, of the antioxidant activity of SGLT2is, which is being corroborated by ever more scientific data. In cell culture conditions, it has been confirmed that empagliflozin very strongly inhibits mitochondrial production of ROS in CMEC. This production is strongly increased by the action of TNF-alpha. Empagliflozin, therefore, by involving intracellular mechanisms, abolishes TNF-alpha-induced overproduction of ROS in CMEC. This leads to a significant reduction in the amount of ROS in the CMEC cytoplasm and to an increase in NO bioavailability, and this, as also previously shown in cell culture conditions, significantly improves the systolic and diastolic function of cardiomyocytes [90].

This has been confirmed in animal studies. In rats with induced diabetes, there was, typically, increased expression of TNF-alpha and INF-gamma as well as increased ROS production. The activation of NOX1 and NOX2 as sources of ROS, disruption of the bioavailability of NO and its signals mediated by cGMP and persistent low-grade inflammation were all confirmed. These studies have shown that all these adverse phenomena are normalised under the influence of empagliflozin, which translates into improved vascular endothelial function. Additionally, in a mouse model of type 2 diabetes, empagliflozin has been shown to reduce the severity of oxidative stress by activating the Nrf2/ARE signalling pathway, which is the primary signalling pathway involved in the regulation of oxidative stress. Empagliflozin promotes the translocation of Nrf2 into the cell nucleus activating the Nrf2/ARE signalling pathway and, thus, reducing oxidative stress in diabetic mice [64]. An additional effect of empagliflozin demonstrated in this study is the reduction of myocardial fibrosis by inhibiting the TGF-Beta1/Smad3 signalling pathway. The inhibition of this signalling pathway has been shown to reduce collagen synthesis in cardiac fibroblasts, reduce myocardial fibrosis and prevent adverse remodelling, when there is pathological left ventricular overload or damage [111–113]. It has been confirmed in the HF animal model that the severity of myocardial inflammation is significantly reduced by inhibiting the TGF-Beta1/Smad3 signalling pathway [114]. In the aforementioned model, high-level activation of the TGF-Beta1/Smad pathway and high expression levels of TGF-Beta1, Smad1, Smad2, Smad3 proteins as well as high levels of type I and III collagen were confirmed in the myocardium of diabetic mice [64]. The use of empagliflozin in these animals significantly reduced the expression of TGF-Beta1, Smad1, Smad2, Smad3 and type I and III collagen levels in their myocardium. In addition, empagliflozin has been shown to significantly increase SMAD7 expression in these animals. This is a known TGF-Beta1 inhibitor and myocardial fibrosis inhibitor [64]. This SGLT2i mechanism of action is particularly interesting, as it has been well documented in animal studies, that inhibition of the TGF-Beta1/Smad3 signalling pathway significantly inhibits myocardial fibrosis and left ventricular remodelling in HF regardless of the presence of diabetes. This may therefore constitute a potential future therapeutic target for SGLT2is in patients with HF, with or without diabetes.

## Conclusions

As outlined above, a new group of antidiabetic drugs, SGLT2is, have various mechanisms of action that go beyond the hypoglycaemic effect. The basic mechanism of action of inhibiting SGLT2 in the proximal tubule of the nephron and thus inducing glucosuria, combined with natriuresis, is, in itself, a unique mechanism of action of these antidiabetic drugs. This gives a hypoglycaemic effect, independent of insulin. However, the uniqueness of this group of drugs was not determined by the strength of their hypoglycaemic activity, but by the very favourable CV outcomes in large, randomised clinical trials. For the first time in history, it has been demonstrated that an antidiabetic drug used in patients with type 2 diabetes and high CV risk significantly reduces CV mortality, all-cause mortality and the number of hospitalisations due to HF. This became a catalyst for a number of studies aimed at understanding the mechanisms of action of this group of drugs that would be able to explain such favourable CV outcomes. As numerous scientific studies have shown, there are many different mechanisms of SGLT2is activity. Some of these do not fully explain these beneficial clinical effects of SGLT2is, especially since in clinical studies devoted to this group of drugs, these benefits occur very early after their introduction. However, other recently recognised mechanisms of SGLT2is action not only allow a better understanding of these drugs’ cardioprotective actions, but, in addition, could be, in the future, a potential therapeutic goal in HF in patients with and without diabetes. As presented above, in some clinical trials a significant percentage of patients had HF with no history of diabetes, and in this group of patients similar clinical benefits were obtained as in those with diabetes. The basic mechanism of action of these drugs also provides the basis for considering this group of antidiabetic drugs as therapy for cardiological indications in patients without diabetes. Their hypoglycaemic effect only occurs in hyperglycaemia, and therefore, there is no risk of hypoglycaemia when used in non-diabetic patients. More research is needed to determine to what extent SGLT2is can be used in HF in non-diabetic patients. The groups of HF patients who would benefit most from this therapy may need to be identified. This would allow for specific indications for the use of SGLT2is in specific groups of patients with HF, depending on its aetiology. The prospect of using this group of drugs in the future seems very attractive, as there is always a need for new treatments that could improve the prognosis in HF.
